# The Diagnostic Role of Novel Echocardiography Indices and Arterial Stiffness in Diabetic Cardiomyopathy

**DOI:** 10.3390/biomedicines13092317

**Published:** 2025-09-22

**Authors:** Elina Khattab, Stefanos Sokratous, Michaela Kyriakou, Georgios Parpas, Ioannis Korakianitis, Paraskevi Papakyriakopoulou, Nikolaos P. E. Kadoglou

**Affiliations:** 1 Medical School, University of Cyprus, Nicosia 2029, Cyprus; khattab_elina@outlook.com (E.K.); sokratous.stefanos@ucy.ac.cy (S.S.); kyriakou.michailia@ucy.ac.cy (M.K.); parpas.georgios@ucy.ac.cy (G.P.); korakianitis.ioannis@ucy.ac.cy (I.K.); 2 Laboratory of Biopharmaceutics-Pharmacokinetics, Department of Pharmacy, School of Health Sciences, National & Kapodistrian University of Athens, 15784 Athens, Greece; ppapakyr@pharm.uao.gr

**Keywords:** diabetic cardiomyopathy, heart failure, diastolic stress echo, myocardial work

## Abstract

**Background/Objectives**: Diabetic cardiomyopathy (DBCM) is characterized by cardiac dysfunction in the absence of ischemic heart disease, hypertension, or valvular disease, often manifesting as heart failure with preserved ejection fraction (HFpEF). Early recognition of DBCM is clinically important, as it enables timely initiation of tailored therapies and may slow down the progression to overt heart failure with reduced ejection fraction (HFrEF). This study aimed to evaluate the diagnostic utility of advanced echocardiographic techniques—myocardial work (MW), diastolic stress echocardiography (DSTE), Cardio-Ankle Vascular Index (CAVI)—and selected serum biomarkers in identifying DBCM. **Methods**: In this prospective observational study with 12-month follow-up, 125 diabetic patients with preserved ejection fraction and symptoms of HF or recent HF hospitalization were enrolled. Using the Heart Failure Association Pre-test Probability of HFpEF criteria, 37 were classified as DBCM-HFpEF and 88 as diabetic controls. An additional 47 age- and sex-matched non-diabetic individuals served as controls. All participants underwent resting echocardiography (MW, GLS), DSTE, CAVI assessment, and biomarker measurement (BNP, troponin, galectin-3). **Results**: Compared to non-diabetics, diabetic patients had significantly higher TRVmax (2.21 vs. 2.05 m/s), LAVI (39.70 vs. 33.50 mL/m^2^), E/e′ (8.64 vs. 7.59), CAVI (8.51 vs. 7.82 m/s), BNP (91.50 vs. 35.10 pg/mL), and troponin (3.94 vs. 2.43 ng/mL) (all *p* < 0.01), while galectin-3 levels showed no significant difference between groups. Differences were more pronounced between DBCM and No-DBCM diabetic groups. Multivariate analysis identified BNP (OR 5.45), TRVmax (OR 8.56), and CAVI (OR 1.91) as independent predictors of DBCM. **Conclusions**: DSTE and CAVI, alongside BNP and echocardiographic parameters, may provide valuable noninvasive tools for the early detection of DBCM in diabetic patients presenting with otherwise unexplained dyspnea, potentially enabling earlier intervention and improved outcomes. This is clinically important guiding an efficient management of an increasing number of diabetic patients presented with unexplained dyspnea.

## 1. Introduction

It is widely known that patients with type 2 diabetes mellitus (T2DM) are characterized by low exercise capacity, despite the co-existence of normal systolic cardiac function [1]. There is an increasing recognition of diabetic cardiomyopathy (DBCM) as the development of cardiac dysfunction and the related heart failure (HF) symptoms when obvious underlying reasons (like myocardial ischemia, hypertension, or valvulopathies) are absent [2]. From an imaging perspective, DBCM may appear with normal left ventricular ejection fraction (LVEF), but in advanced stages may progress to heart failure with reduced ejection fraction (HFrEF). The underlying pathophysiology of DBCM is complex, involving a combination of metabolic, structural, and functional alterations. Chronic hyperglycemia, insulin resistance, and lipotoxicity contribute to oxidative stress, mitochondrial dysfunction, and maladaptive inflammatory responses. These processes result in early myocardial stiffening, diastolic dysfunction, and left atrial (LA) impairment, ultimately progressing to fibrosis, fatty infiltration, and adverse left ventricular (LV) remodeling [3]. Importantly, cardiac autonomic neuropathy (CAN) has emerged as a crucial mechanism, as autonomic imbalance impairs myocardial perfusion, alters diastolic relaxation, and predisposes to arrhythmia, further aggravating cardiac dysfunction in diabetic patients [4].

Regarding the high frequency of HF-related symptoms among an otherwise “cardiac-disease free” diabetic population and the adverse prognosis of HF, an early detection and tailored treatment of HF in diabetic patients is deemed necessary. After ruling out common reasons for HF, the current criteria for DBCM diagnosis comply with those from either HFpEF or HFrEF algorithms, where the former is more challenging [5]. Except for diastolic dysfunction grade III, in the rest of DBCM cases with preserved LVEF, a cluster of resting echocardiography parameters and B-type natriuretic peptide (BNP) levels is required to document increased left ventricular filling pressures (LVFP). In most cases, the clinical manifestations of LVFP, dyspnea, and fatigue become apparent during an exercise test [5]. Previously, a study group from the European Society of Cardiology proposed the HFA-PEFF (Heart Failure Association Pre-test Probability of HFpEF) score for HFpEF diagnosis. Alongside speckle tracking analysis (STA) and natriuretic peptide assays, diastolic stress test echocardiography (DSTE) has also been incorporated into the score, providing a more sensitive assessment in cases where resting echocardiography and natriuretic peptide levels suggest only subtle cardiac dysfunction [6,7]. DSTE is a cheap, easily performed, and repeatable test that can uncover exercise-induced elevation of LVFP linked to exertional dyspnea [8].

Global longitudinal strain (GLS) has long been demonstrated as a representative index of STA to assess systolic dysfunction with high prognostic value among the diabetic population [9]. Recently, myocardial work (MW) represents a novel imaging parameter, combining GLS and blood pressure to calculate the pressure–strain loop (PSL) of LV [10]. MW indices include global myocardial work index (GWI) for total performance, global constructive work (GCW) for effective energy use, global wasted work (GWW) for inefficiency of systolic contraction, and global work efficiency (GWE) for energy efficiency. In T2DM, reduced GWI and GWE and increased GWW indicate early myocardial dysfunction [10].

Besides echocardiographic indices, arterial stiffness has long been associated with increased cardiovascular risk in T2DM [11]. Reduced elasticity and compliance of large arteries have also been increasingly recognized as a contributing factor for the development of diastolic dysfunction in HFpEF, affecting early systolic pressure rise and the timing of arrival of wave reflections to the LV [12,13]. The most common functional parameter of arterial stiffness is pulse wave velocity (PWV) [13]. Recently, the cardio-ankle vascular index (CAVI) has gained attention due to its independence of blood pressure (BP), contrary to PWV [14]. CAVI is calculated based on stiffness parameter β, which incorporates systolic and diastolic BP, heart-ankle PWV, and pulse wave characteristics derived from ECG and phonocardiography signals [5]. Higher CAVI scores have been associated with increased risk for composite cardiovascular events in high-risk populations [15] and have been independently associated with diastolic dysfunction in HFpEF [16]. These findings support the potential utility of CAVI for DBCM diagnosis. There is also a growing interest in circulating biomarkers such as B-type natriuretic peptide (BNP), high-sensitivity troponin (hsTn), and galectin-3. They hold promise in detecting early HF by identifying high intracardiac pressure, fibrosis, inflammation, and cardiac remodeling [17]. Those circulating biomarkers may enable early recognition and efficient management of DBCM, preventing progression to HFrEF.

In our study, we hypothesized that novel echocardiography parameters, CAVI, and biomarkers may be involved in HFpEF diagnosis in T2DM patients with unexplained exertional dyspnea and/or fatigue at early stages. We aimed to assess the potential relationship between CAVI, GLS, myocardial work (MW) indices, and biomarkers (BNP, hsTn, galectin-3) with the presence of DBCM in patients with preserved LVEF.

## 2. Materials and Methods

### 2.1. Study Design

This is a clinical, prospective, observational, non-randomized trial with a 12-month follow-up in patients with T2DM. The study was conducted between January 2022 and January 2025. The presence of T2DM was confirmed based on fasting plasma glucose (FPG) levels of ≥126 mg/dL and hemoglobin A1c (HbA1c) levels of ≥6.5% measured within the last three months or in patients already receiving anti-diabetic treatment. Among a large pool of 286 T2DM patients, we enrolled those who met at least one of the following two conditions: (1) HF symptoms, such as dyspnea and/or fatigue for at least 3 months, which remained unexplained despite prior diagnostic evaluation; (2) history of hospitalization for acute dyspnea, where a diagnosis of HFpEF was set.

At baseline, a cohort of 42 non-diabetic individuals without HF symptoms (3:1 ratio) matched for age, gender, hypertension, and dyslipidemia prevalence served as non-diabetic controls of the T2DM patients.

All participants underwent an extensive investigation before entering the study like medical history recording (medications and chronic comorbidities) and lab tests conducted within the last year. Primarily, all participants had LVEF > 50% on echocardiography, and the coronary artery disease (CAD) was excluded by functional tests negative for myocardial ischemia or coronary angiography. Besides this, there was no evidence of at least moderate valvular diseases or significant peripheral artery disease, based on ultrasound examinations. We also excluded patients with the inability to perform DSTE (e.g., orthopedic issues), suboptimal acoustic windows, diastolic dysfunction grade III at rest, severe chronic co-morbidities causing HF-like symptoms, and negatively affecting exercise capacity and/or prognosis (e.g., chronic kidney or liver disease, diabetic neuropathy, active malignancy, autoimmune diseases, untreated thyroid disorders, chronic anemia). Patients with clinically significant diabetic neuropathy were excluded based on neurological examination and clinical history (e.g., presence of peripheral sensory loss, neuropathic pain, or positive autonomic dysfunction tests). Although patients with DBCM may have a predisposition to neuropathy, we limited inclusion to those without clinically manifest neuropathy.

After obtaining a written informed consent, all eligible participants underwent a comprehensive evaluation, including the following: (1) clinical examination (body mass index, blood pressure), (2) arterial stiffness assessment via CAVI measurement (3) resting echocardiography and STA with calculation of GLS, and MW indices, (4) DSTE, (5) laboratory testing for glycemic and lipid profiles, renal and liver function, hs-Tn and HF-related biomarkers such as BNP and galectin-3. A detailed description of all measurements is provided in the next sections.

After baseline evaluation, the diagnosis of DBCM with preserved LVEF was made in T2DM patients who achieved HFA-PEFF score ≥ 5 [18]. Accordingly, diabetic patients were stratified into two groups: (1) Patients with DBCM and (2) Patients with No DBCM. All patients with DBCM received the optimum medical therapy for symptom relief (mainly diuretics) and SGLT2i for prognosis improvement, adapted to precautions and contraindications. Follow-up lasted 12 months, during which we recorded any new episode of dyspnea requiring either hospital admission as an acute HF or up-titration of diuretics and other pharmaceutical agents for HF-related symptoms relief. Except for the biomarker assay, the whole evaluation process was repeated at the end of the follow-up period in the DBCM and No-DBCM groups.

All participants were recruited consecutively after an open invitation from the Medical School, University of Cyprus. They were thoroughly informed about the study’s nature, scope, and requirements through personal consultations, and they provided informed consent prior to study entry. The study protocol complied with the Helsinki Declaration and was approved by the National Bioethics Committee (NCBC 2022/52) [19].

### 2.2. Resting Echocardiography

Echocardiography was performed using commercially available echocardiographic systems, and image analyses were conducted on workstations by two trained cardiologists (NK, ΕΚ). LVEF was calculated using Simpson’s method. Relative wall thickness: RWT = (2 times {inferolateral wall thickness})/{LV internal dimension}, with LV concentric remodeling defined as RWT > 0.42. LV mass was calculated using the linear method, and LV hypertrophy was defined as a left ventricular mass index (LVMI) exceeding 95 g/m^2^ for women and 115 g/m^2^ for men. Trans-mitral pulsed-wave Doppler and tissue Doppler imaging were acquired from the apical four-chamber view to measure early (E) and late (A) diastolic flow velocities, as well as early diastolic (e′) velocities of the mitral annulus at the septal and lateral walls. The E/e′ ratio was calculated as the average of the septal and lateral site measurements. LA volumes were obtained using the biplane area-length method.

LV diastolic function was assessed at rest by combining pulsed-wave Doppler evaluation of mitral inflow and tissue Doppler imaging of the mitral annulus. Diastolic function was classified into the following categories: normal (0.75 < E/A < 1.50 and E/e′ < 10); grade I, defined as impaired relaxation without evidence of increased filling pressures (E/A ≤ 0.75 and E/e′ < 10); grade II, defined as impaired relaxation with moderate elevation of filling pressures or pseudonormal filling (0.75 < E/A < 1.50 and E/e′ ≥ 10); and grade III, defined as advanced reduction in compliance with reversible or fixed restrictive filling (E/A > 1.50 and E/e′ ≥ 10).

### 2.3. Diastolic Stress Echocardiography (DSTE)

All participants underwent DSTE using a supine ergocycle (Ergoline ergoselect 12, Bitz, Germany). Even in patients achieving an HFA-PEFF score ≥ 5, we proceeded to DSTE. The exercise workload was incrementally increased by 25 watts every 2 min following the World Health Organization protocol For participants who encountered difficulty maintaining cycling rate, a modified protocol with an initial workload of 10 watts and subsequent increases of 10 watts per 2-min was used. The parameters were recorded during DSTE at a heart rate of up to 100–110 bpm before fusion of E and A waves. The tests were of submaximal workload, and symptoms appearing during the test were documented. In addition to the latter parameters, we also assessed early diastolic tissue velocity (E′) at the medial and lateral mitral annulus, tricuspid regurgitation maximum velocity (TRVmax), and cardiac output (calculated as stroke volume multiplied by heart rate). Pulmonary artery systolic pressure (PASP) was calculated using the simplified Bernoulli equation, based on the TRVmax and the right atrial pressure, which was estimated from the inferior vena cava imaging at rest.

### 2.4. Global Longitudinal Strain (GLS) and Myocardial Work (MW)

The measurement of global longitudinal strain of LV provides a detailed assessment of LV myocardial deformation by tracking natural acoustic markers on a frame-to-frame basis throughout the cardiac cycle. Longitudinal strain, which evaluates the shortening and lengthening of the myocardial wall, was measured from three apical views: the 2-chamber view (assessing anterior and inferior walls), the 4-chamber view (posteroseptal and lateral walls), and the 3-chamber view (anteroseptal and posterior walls). Each wall was divided into three regions (basal, mid, and apical), generating a total of 17 segmental strain curves. During measurement, the regions of interest were manually adjusted to include the entire myocardial thickness while avoiding simultaneous inclusion of the pericardium. GLS was calculated as the average of the peak strain values from the three apical views. The intra- and inter-observer variability of strain analysis within the study team has been previously validated (<3%). Additionally, myocardial work at rest was calculated by incorporating blood pressure measurements into GLS analysis based on the manufacturer’s software.

### 2.5. Cardio-Ankle Vascular Index (CAVI) Measurement

CAVI was measured using the vascular screening system VaseraVS-1500 (Fukuda Denshi, Tokyo, Japan). We initially measured height (in cm) and weight (in kilograms), which were inserted into the algorithm, along with the age and sex of each participant. All participants were then placed in a supine position for approximately 10 min, during which time ECG electrodes were placed on both wrists, along with a system of heart sound recording on the sternum. In addition, four cuffs with pulse sensors were placed on each of the four limbs. The pulse wave velocity was measured from the aorta to the distal end of each tibial artery (ankle), while at the same time taking measurements of systolic and diastolic BP at the left brachial artery. Two measurements were taken for each side (right upper and lower extremities, left upper and lower extremities). These measurements were then inserted into the algorithm that calculates CAVI, a representation of arterial stiffness, as was suggested by Hayashi, by applying Bramwell–Hill’s ratio. Specifically, CAVI is calculated by the equation: CAVI = aβ + b, where a and b are constants, and β is the arterial stiffness parameter. Substituting for β, the equation becomes CAVI = a [(2ρ/ΔΡ × In (Ps/Pd) × PWV2)] + b, where ρ is blood density, ΔΡ is systolic pressure (Ps) minus diastolic pressure (Pd), measured in mmHg, and PWV is from the aorta to the tibial artery. CAVI is measured in arbitrary units, and an abnormal value > 9 indicates increased arterial stiffness [15].

### 2.6. Blood Measurements

All patients underwent blood testing for the measurement of BNP and galectin-3 only at baseline. Blood samples were promptly collected after overnight fasting and were subjected to centrifugation. The resulting serum was stored at −80 °C in a deep freezer. The measurements of serum troponin (high sensitivity) and BNP were conducted using the Alinity analyzer from Abbott Diagnostics (Abbott Park, Libertyville Township, IL, USA). This process involved a two-step immunoassay conducted in human serum, utilizing chemiluminescent microparticle immunoassay technology. According to the manufacturer’s specifications, the precision of the troponin and BNP assay at low concentrations is adequate, enabling the assessment of various thresholds with a coefficient of variation (CV) of 3.2% in our laboratory. Serum concentrations of Galectin-3 (GAL3) were determined using a commercially available enzyme-linked immunosorbent assay (ELISA) kit (FineTest^®^, EH0145, Wuhan Fine Biotech Co., Ltd., Wuhan, China), following the manufacturer’s instructions. Serum samples (50 μL per well) were incubated with biotin-labeled detection antibody and horseradish peroxidase (HRP)-conjugated streptavidin. The signal was developed using a tetramethylbenzidine (TMB) substrate and quantified spectrophotometrically at 450 nm. All serum samples were processed and stored at −80 °C until analysis. The intra-assay coefficient of variation (CV) ranged from 4.23% to 5.22%, and the inter-assay CV ranged from 5.02% to 5.43%, based on internal quality control samples with low, medium, and high GAL3 concentrations. All measurements were performed in duplicate, and standard curves were constructed. Blood sample analysis was performed at the Medical School, University of Cyprus.

### 2.7. Statistical Analysis

Normal distribution of all variables was initially assessed. Continuous variables were expressed as mean ± standard deviation (SD) for normally distributed data or as median and interquartile range (IQR) for skewed data. Comparisons between DBCM and No-DBCM patients were performed using either Student’s *t*-test for continuous variables or the chi-square test. At baseline, one-way ANOVA and Kruskal–Wallis test with post hoc analysis were used for the comparative evaluation between three groups for continuous and categorical variables, respectively. Correlations were assessed using Pearson’s correlation coefficient (R) or Spearman’s rho, depending on data distribution. Multiple regression analysis was conducted to identify independent predictors of DBCM diagnosis within the diabetic population. A two-tailed *p*-value < 0.001 was considered statistically significant. Statistical analyses were performed using SPSS software (version 29.0; IBM Corp., Armonk, NY, USA).

## 3. Results

### 3.1. Study Population

We initially selected 141 patients fulfilling at least one of the two conditions. After initial investigation, a total of 125 diabetic patients and 42 non-diabetic individuals were enrolled. Then, diabetic participants were categorized into two groups: diabetic patients with DBCM based on HFpEF diagnostic criteria and evidence of diastolic dysfunction (DBCM group, *n* = 37) and diabetic patients without HFpEF (No-DBCM group, *n* = 88). Demographic and clinical characteristics did not vary between groups as depicted in Table 1. The presence of men (56.90%) was slightly higher than women. The non-diabetic group tended to be older than the diabetic group, but this was not statistically significant (*p* = 0.115). Most diabetic patients manage their condition with oral antidiabetic tablets, while the rest use insulin injections. No statistically significant differences between groups were detected regarding body mass index (BMI) (*p* = 0.088), hypertension (*p* = 0.300), and dyslipidemia (*p* = 0.787) prevalence. Only one quarter of the participants were smokers. None of the participants had significant peripheral artery disease as expressed by the brachial-ankle index.

Lastly, CAVI values were found significantly lowered across diabetic groups vs. control group. Within the diabetic cohort, the DBCM patients had even higher CAVI values than their No-DBCM counterparts (*p* = 0.023).

### 3.2. Echocardiographic Findings

A detailed comparison of echocardiographic parameters between all groups did not reveal significant differences in LVEF (*p* = 0.968) and GLS (*p* = 0.790) values, indicating the absence of systolic impairment, even at a subclinical level, in the diabetic groups (Table 2). Moreover, non-significant differences in GLS and all indices of myocardial work (Global Work Index—GWI; Global Work Efficiency—GWE; Global Constructive Work—GCW; Global Wasted Work—GWW) were detected among groups. The left atrium volume index (LAVI) was significantly higher in the DBCM group (39.71 ± 8.27 mL/m^2^) compared to No DBCM (34.46 ± 9.50 mL/m^2^, *p* = 0.042) and non-diabetic controls (32.64 ± 7.21 mL/m^2^, *p* < 0.001). Only the E/e′ ratio, a surrogate for LVFP, was significantly higher in the DBCM than the No-DBCM group (*p* = 0.020) and non-diabetic controls (*p* < 0.001), while the E/A ratio did not differ between groups. In terms of right ventricular (RV) function, the RV systolic velocity (RV S’) and the tricuspid annular plane systolic excursion (TAPSE) did not show statistically significant differences (*p* > 0.050).

### 3.3. Diastolic Stress Echocardiography

We performed DSTE in all DBCM patients, even though 28 of them had already achieved an HFA-PEFF score ≥ 5 before the test. Our aim was to test how the results of DSTE fit with the HFpEF diagnosis in diabetic patients. Unfortunately, among DSTE criteria, only 3 patients achieved E/e′ > 15, and 21 patients achieved TRVmax > 3.4 m/s. However, all DBCM patients reported dyspnea during the test, and one quarter of them asked to stop the test or were unable to continue cycling due to fatigue. In the case of No DBCM, none of them achieved E/e′ > 15, and three patients achieved TRVmax > 3.4 m/s during DSTE, but they did not achieve a score ≥ 5. Dyspnea or fatigue during DSTE was reported by only four No-DBCM patients, but none of them stopped the test prematurely. As expected, control individuals did not report any symptoms in their low workload DSTE.

During DSTE, the E/A ratio tended to be lower in DBCM vs. No-DBCM patients (*p* = 0.075), while there was a significant difference in the E/e′ ratio between groups (DBCM vs. No DBCM, *p* < 0.001), indicating elevated LVFP under stress (Table 2). Both groups showed similar levels of the TRVmax at rest (*p* = 0.064), but TRVmax was remarkably elevated during submaximal DSTE in the DBCM group (from 2.23 ± 0.29 to 2.91 ± 0.49 m/s) compared to No DBCM (from 2.03 ± 0.27 to 2.33 ± 0.29 m/s, *p* < 0.001), highlighting a considerable rise in pulmonary pressure under exertion. Additionally, the average increase in TRVmax as a percentage of baseline value was 25% among DBCM patients and 15% in their No-DBCM counterparts (*p* < 0.001).

All DBCM patients achieved HFA-PEFF score ≥ 5, which was mainly driven by the high LAVI and the elevated BNP levels (values fulfilling major criteria). In particular, both diabetic groups had larger LA volume than controls, while it was further exaggerated in the DBCM group (39.71 ± 8.27 mL/m^2^) compared to No DBCM (34.46 ± 9.50 mL/m^2^, *p* = 0.042). With the exception of LAVI, the No-DBCM and non-diabetic group did not differ in any of the other resting echocardiography and DSTE parameters.

### 3.4. Follow-Up Results

Three patients were lost during the follow-up period, while 122 patients repeated the initial evaluation with resting echocardiogram, DSTE, and blood sampling (Table 3). All patients in the DBCM group received optimal medical therapy including any combination of the following classes of drugs when tolerated: SGLT-2 inhibitors (empagliflozin or dapagliflozin), ACE inhibitors, diuretics, b-blockers, and eplerenone (Table 4). The former class of drugs was prescribed for the majority of patients with a DBCM diagnosis. At the same time, anti-hypertensive and anti-diabetic medical therapy were intensified in the No-DBCM group. During the 12-month follow-up, two patients in the DBCM group were admitted with acute decompensated heart failure requiring intravenous diuretics, while no hospitalizations occurred in the No-DBCM group. Additionally, nine patients with DBCM reported an improvement of at least one NYHA functional class, whereas the rest did not mention any change in functional capacity despite optimized therapy. In the No-DBCM group, most patients remained stable, with seven showing modest functional improvement after intensification of anti-diabetic and anti-hypertensive therapy. No deaths or major cardiovascular events (e.g., myocardial infarction, stroke) were recorded in either group.

### 3.5. Biomarkers

**Figure 1:**
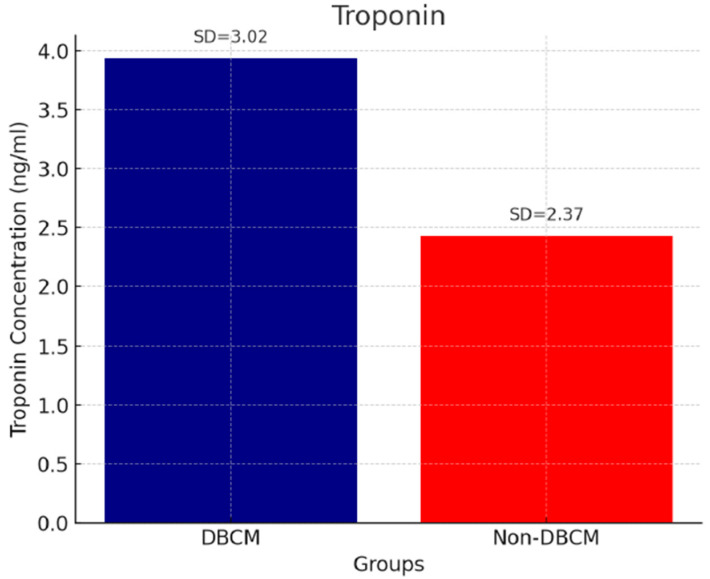
Troponin and DBCM.

**Figure 2:**
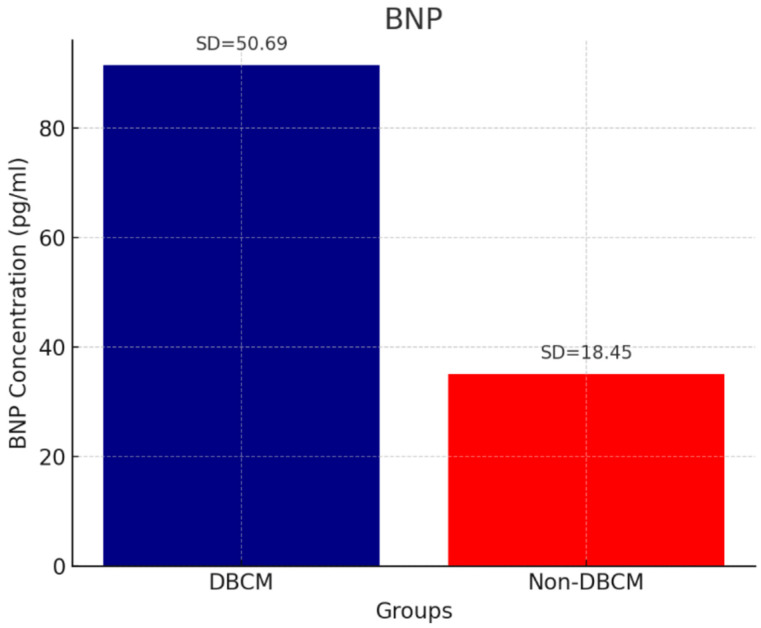
BNP and DBCM.

**Figure 3:**
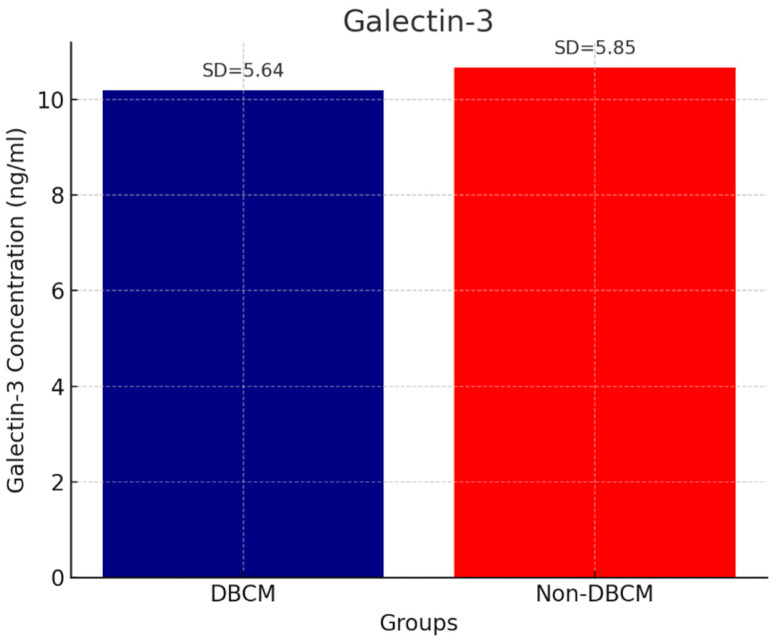
Galectin-3 and DBCM.

Analysis of circulating cardiac biomarkers revealed a significantly higher burden of myocardial stress and fibrosis in the DBCM group. Troponin levels remained within normal range, but they were higher in the DBCM group (3.94 ± 1.02 ng/mL) compared to the No-DBCM group (2.43 ± 1.37, *p* = 0.013) (Figure 1). As expected, we found significantly higher levels of BNP in the DBCM (91.48 ± 50.69 pg/mL) versus No-DBCM (35.10 ± 18.45 pg/mL, *p* < 0.001) group and non-diabetic group (15.01 ± 5.98 pg/mL, *p* < 0.001), reflecting increased cardiac wall stress or volume overload (Figure 2). Finally, the difference between groups in galectin-3 levels did not reach statistical significance (*p* = 0.691) (Figure 3).

### 3.6. Correlations

In univariate analysis, the presence of DBCM was correlated with E/e′, LAVI, TRVmax, CAVI, troponin, and BNP at baseline and TRVmax and E/e′ during DSTE. In logistic regression analysis, the BNP, TRVmax at rest, and TRVmax during DSTE remained independent determinants of DBCM diagnosis (Table 5).

## 4. Discussion

### 4.1. Overall Findings

To our knowledge, this is the first study that evaluated the role of DSTE, speckle tracking, and CAVI in DBCM diagnosis. The main findings of this prospective, non-randomized study in diabetic patients with unexplained dyspnea and/or fatigue but without ischemic or valvular disease were as follows: (1) Patients with DBCM had significantly higher LAVI, TRVmax at rest and during stress test than No-DBCM patients and non-diabetic controls. (2) Those findings, along with the 3-fold higher BNP levels, were independently associated with DBCM diagnosis. (3) Arterial stiffness as expressed by CAVI was significantly elevated in diabetic vs. non-diabetic counterparts, but most importantly, it was independently associated with DBCM diagnosis, reinforcing its clinical relevance. (4) The novel modality of speckle tracking (GLS and myocardial work indices) did not distinguish DBCM from No-DBCM patients. (5) Our study was underpowered to assess any association of DBCM with clinical outcomes.

### 4.2. Echocardiographic Indices (GLS, Myocardial Work, and LAVI)

It is well known that LVEF may remain within normal limits even when there is significant impairment in myocardial deformation. This highlights the need for more sensitive tools to detect early cardiac dysfunction in diabetic patients. Our study assessed LV function in patients with T2DM, combining the novel, non-traditional parameters like MW analysis, biomarkers, and DSTE for diastolic dysfunction. LVEF was preserved in all diabetic participants, and there was no significant difference from non-diabetic controls. Moreover, novel echocardiographic parameters, like GLS and MW indices, failed to distinguish DBCM from No-DBCM and non-diabetic controls. Differences in GWI, GCW, GWE, and GWW tended to favor No DBCM, but they did not reach statistical significance. Huang et al. demonstrated that T2DM patients had significantly lower GWI and GWE compared to healthy controls, indicating early myocardial impairment despite normal LVEF [20]. Similarly, other studies reported decreased GLS, GWI, and GCW, along with increased GWW, in T2DM patients, further supporting the utility of STA and MW parameters in detecting subclinical myocardial dysfunction [21,22]. However, all previous studies have compared T2DM patients with healthy controls. In our study, the diabetic cohort had a similar cardiovascular risk profile (e.g., hypertension, dyslipidemia) with non-diabetic controls. Based on our findings, GLS and MW indices may not be significantly affected by the presence of T2DM. Moreover, they may not effectively apply to identify diabetic patients with HFpEF. Presumably, diastolic dysfunction alone does not significantly affect these novel echocardiographic indices when systolic function is preserved or only mildly impaired. Importantly, while previous studies demonstrated lower absolute absolutGLS and abnormal myocardial work indices in T2DM compared to healthy individuals, our findings indicate that these parameters could not discriminate diabetic patients with DBCM from those without. This apparent ‘negative′ result carries clinical significance: it highlights that although GLS and MW indices are valuable for detecting subclinical systolic dysfunction in asymptomatic patients, they may be insufficiently sensitive or specific for identifying symptomatic diabetic patients with HFpEF. In clinical practice, this underscores the need for a multimodal diagnostic approach to diastolic function which integrates additional indices such as DSTE with TRVmax measurements, biomarkers like BNP, and vascular markers such as CAVI, rather than relying solely on speckle tracking indices.

The high prevalence of LA enlargement in our DBCM cohort, along with BNP elevation, remarkably increased the calculated HFA-PEEF score, contributing to HFpEF diagnosis. Instead of LA antero-posterior diameter, we used the LAVI marker. It was markedly increased in our DBCM group, reflecting an LA overload and presumably impaired LA function, usually the reservoir and booster pump function. These changes mirror the chronic hemodynamic burden imposed by diastolic dysfunction and contribute to the clinical manifestations of HFpEF. The dilatation of LA has been associated with poor prognosis in HFpEF and should always be monitored during the follow-up of those patients [23]. An interplay between atrial myopathy and HFpEF has been proposed, which may contribute to HFpEF development and progression. However, that was not the point of our study, and the LA function should be evaluated in the future [24].

### 4.3. Arterial Stiffness

Arterial stiffness, assessed by the CAVI in our study, has been previously shown to be increased in individuals with diabetes [10]. Elevated CAVI values have also been associated with diastolic dysfunction and, importantly, a higher risk for major adverse cardiovascular events (MACE) in various high-risk populations [12,25]. Notably, Osawa et al. demonstrated a significant correlation between increased CAVI and LAVI in young adults with suspected CAD, predominantly diabetic and hypertensive, supporting the link between arterial stiffness and atrial remodeling across different clinical contexts [26]. In T2DM, Kim et al. reported that elevated CAVI values are associated with the presence of microvascular complications such as neuropathy and nephropathy, predominantly driven by hyperglycemia [27]. Our study extends this to the specific context of DBCM, demonstrating that CAVI was not only significantly higher in diabetic patients and even exaggerated in patients with DBCM compared to No-DBCM counterparts. One of the major findings of our study was the independent association of CAVI with DBCM diagnosis, reinforcing its clinical relevance as a diagnostic tool, while underscoring also the detrimental effects of insulin resistance and chronic hyperglycemia on vascular structure and function [13,14]. Additionally, the parallel increases in markers of diastolic dysfunction as observed in the DBCM group support the interplay of arterial stiffness with early myocardial stiffening and impaired relaxation in DBCM. Our findings highlight the potential of CAVI as a practical, non-invasive marker for early detection and risk stratification in the diabetic population with HFpEF.

### 4.4. Biomarkers

As expected, our DBCM group showed significant elevation of circulating biomarkers of myocardial injury and wall stress due to volume overload. While some studies failed to demonstrate a statistically significant association between BNP levels and DBCM, others have reported that elevated BNP levels correlate with clinical progression of the disease [28,29,30]. Natriuretic peptides remain the cornerstone for HF diagnosis in the general population or specific subpopulation, despite the existence of several limitations [31]. In our cohort, DBCM patients presented with almost three times higher BNP levels than their No-DBCM counterparts, showing a high discrimination power for HF diagnosis. The cut-off value of BNP may be reconsidered in diabetic populations, regarding the frequent comorbidities which may influence those circulating levels. In our study, troponin concentrations remained within normal range, but it was markedly higher in DBCM. On the other hand, the difference in galectin-3, a fibrosis-related marker, between groups did not reach statistical significance. The current literature remains inconclusive for the latter two biomarkers. The observed ‘normal’ fluctuation of troponin may indicate a subclinical myocardial injury but needs further investigation. The ARISE-HF trial demonstrated an association of elevated troponin levels with impaired diastolic relaxation and reduced absolute GLS, suggesting subclinical myocardial injury in diabetic patients [30]. Moreover, it has been documented that there is a higher risk for adverse events in patients with HFpEF and elevated troponin levels [32]. In parallel, galectin-3 has been recognized as a valuable biomarker of HFrEF, but not for HFpEF [33]. It may be elevated in HFpEF patients after the development of LV systolic dysfunction and adverse remodeling [34]. Recent studies have demonstrated that galectin-3 levels are significantly associated with the diagnosis and prognosis of DBCM patients, as elevated levels correlate with impaired cardiac function, reduced absolute GLS, and an increased risk of cardiovascular events and mortality in individuals with T2DM and reduced LVEF [35,36,37]. Galectin-3 is known to reflect myocardial fibrosis and remodeling—key pathophysiological processes in DBCM—and has shown prognostic associations with both systolic/diastolic dysfunction and cardiovascular outcomes among individuals with T2DM [38,39]. In our study, all patients had preserved LVEF (>50%), which may explain the absence of significant elevation of galectin-3 in our DBCM group. Elevated troponin, while nonspecific, indicates ongoing subclinical myocardial injury and has prognostic value across the HF spectrum. As noted in recent reviews, conventional markers like natriuretic peptides and troponins alone are limited in detecting early DCM, especially in asymptomatic stages [17]. The role of troponin and galectin-3 in DBCM diagnosis should be further evaluated because they retain potential utility within a multimarker framework. Emerging evidence supports such an approach including multiple biomarkers—galectin-3, soluble suppression of tumorigenicity-2 (sST2), growth differentiation factor-15 (GDF-15), fibroblast growth factor-21 (FGF-21), and insulin-like growth factor-binding protein-7 (IGFBP-7)—alongside natriuretic peptides to improve diagnostic accuracy and risk stratification in diabetic patients [17]. For example, galectin-3 combinations with NT-proBNP have enhanced risk prediction in HF populations [40]. Therefore, while our data for galectin-3 and troponin alone do not reach statistical significance, they can still add meaningful pathophysiological and prognostic context when integrated into a comprehensive biomarker panel.

### 4.5. Diastolic Stress Echocardiography

While DSTE is a recommended tool for HFpEF diagnosis, when resting findings are inconclusive, its application in diabetic patients has been limited [17]. A recent study by Alseenmy A showed that patients with T2DM exhibited impaired LV diastolic reserve, as evidenced by a significantly higher post-exercise septal E/e′ ratio and a reduced septal e′ velocity compared to non-diabetic individuals [41]. The present study is innovative in expanding the DSTE application to all diabetic participants complaining of HF symptoms, irrespective of HFA-PEFF score achieved at rest. Incorporating the key exercise-based parameters—E/e′ ratio and TRVmax—we evaluated the level of agreement between them and HFpEF diagnosis. Their contribution to the HFA-PEFF score was not as high as we expected, since among our DBCM patients, 3 achieved E/e′ > 15 (giving 2 points) and 21 achieved TRVmax ≥ 3.4 m/s (giving 1 more point) during DSTE. Most of our DBCM patients (28 out of 37) already fulfilled the diagnostic criteria for HFpEF at rest, but 11 of them had normal DSTE findings. The latter finding of ‘normal’ DSTE findings in DBCM patients questioned whether the used cut-off values of the aforementioned parameters add significant value to HFpEF diagnosis. Although E/e′ ≥ 15 has been proposed as a representative index of elevated LVFP during exercise stress, we hypothesized that a lower absolute TRVmax value and the amount of change in TRVmax may point toward rapid elevation of pulmonary artery pressures because of high LVFP in HFpEF. We also hypothesized that TRVmax ≥ 2.9 m/s during DSTE may fit better with diastolic dysfunction leading to higher pulmonary pressure. A noteworthy finding in our study was the discrepancy between resting HFA-PEFF scores and DSTE results. Specifically, 11 patients with DBCM fulfilled HFpEF diagnostic criteria at rest but displayed ‘normal’ DSTE findings when conventional cut-offs (E/e′ ≥ 15 or TRVmax ≥ 3.4 m/s) were applied. This discordance raises important questions about the sensitivity of current DSTE thresholds in diabetic populations. It is possible that the pathophysiology of DBCM involves earlier or smaller amounts of changes in pulmonary pressures and diastolic reserve that are not adequately captured by traditional criteria. In fact, our data showed that relative increases in TRVmax (ΔTRVmax ≥ 25%) during exercise were common in DBCM patients and may be more sensitive than absolute thresholds. Thus, normal DSTE in this setting should not be interpreted as excluding HFpEF, but rather as evidence that conventional cut-offs may underestimate early diastolic dysfunction in T2DM. These findings suggest that the clinical role of DSTE in diabetes may lie not only in confirming HFpEF when resting data are equivocal, but also in guiding refinement of diagnostic criteria tailored to this high-risk population. These findings underscore the need for further research using standardized DSTE protocols to enhance early detection and risk stratification in this high-risk population. Multivariate analysis identified CAVI, BNP, TRVmax at rest, and TRVmax during DSTE as independent predictors of DBCM diagnosis. These parameters reflect both central and peripheral components of cardiac dysfunction—CAVI depicting high vascular resistance, BNP capturing myocardial wall tension, and TRVmax reflecting pulmonary vascular load—making them promising markers for prompt diagnosis and prognosis in diabetic patients. Although our study was not designed to assess the efficacy of therapeutic interventions, we believe that the routine combined usage of echocardiography, DSTE, CAVI, and biomarkers in clinical practice may improve clinical outcomes in patients presented with HF symptoms. Also, although the study was not powered for prognostic endpoints, the 12-month follow-up provides preliminary insights into clinical outcomes. The occurrence of acute HF hospitalizations exclusively in the DBCM group and the more frequent NYHA class improvement in patients receiving SGLT2 inhibitors suggest that early identification and tailored therapy may improve symptoms and reduce adverse events. Nevertheless, larger and longer-term studies are required to validate the prognostic implications of these findings.

### 4.6. Follow-Up and Prognostic Implications

Our 12-month follow-up provided preliminary insights into the prognostic implications of DBCM. Two patients in the DBCM group required hospitalization for acute decompensated HF, while no hospitalization occurred in the No-DBCM group. Moreover, a subset of DBCM patients reported functional improvement after initiation of SGLT2 inhibitors. Due to limited event rate, sample size, and duration, these findings suggest that DBCM may be at higher risk of adverse outcomes and that timely therapeutic intensification should be committed to reducing hospitalizations. Future larger longitudinal studies are required to validate the prognostic impact of these parameters.

### 4.7. Limitations and Future Directions

This study has several limitations that must be acknowledged. First, the sample size, particularly of the DBCM group, was relatively small, which may limit statistical power in detecting subtle differences in certain parameters such as galectin-3, GLS, or myocardial work indices. The sample size of DBCM was less than 50% of the control group, which raises the possibility of Type II error in achieving true associations with statistical significance. Therefore, our negative findings in these parameters should be interpreted with caution and confirmed in larger, adequately powered studies. Second, the echocardiographic analysis relied on operator-dependent measurements, and although consistency was maintained through standardized protocols, inter-observer variability was not formally assessed. Thirdly, the workload achieved during DSTE was low which might not fully capture exertional limitations or hemodynamic responses. On the other hand, a rapid increase in TRVmax at an early stage of stress echocardiography indicated high vascular resistance and has been demonstrated to be a significant determinant of future events [42]. Lastly, although follow-up was conducted, the duration was relatively short, the pharmaceutical changes were decided by patients’ physicians, and the long-term prognostic implications of the observed findings remain to be clarified in larger prospective cohorts.

## 5. Conclusions

In the present study, we integrated conventional echocardiography, DSTE, STA, CAVI, and circulating cardiac biomarkers. We tailored a global approach to myocardial dysfunction associated with DBCM. We demonstrated that elevated BNP levels as well as TRVmax at rest and during exercise and dilated LA mainly contributed to DBCM diagnosis, in a diabetic cohort with preserved LVEF and unexplained dyspnea and/or fatigue. Notably, DBCM patients showed a considerable elevation in CAVI than No-DBCM patients. The novel STA indices of GLS and myocardial work were not affected by the DBCM. This study underscores the utility of a cluster of parameters to uncover diastolic dysfunction and hence HFpEF in diabetic patients. The novelty of our study lies not only in demonstrating the diagnostic utility of DSTE, BNP, and CAVI in diabetic patients with HFpEF, but also in showing that GLS and myocardial work indices—despite their value in subclinical disease—may not provide sufficient discriminatory power in this clinical context. This negative finding is important for guiding clinicians away from overreliance on speckle tracking indices alone and toward a more comprehensive, multimodal diagnostic strategy. Future research with larger, longitudinal cohorts is warranted to validate these findings, evaluate their prognostic implications, and explore the clinical impact of personalized treatment strategies.

## Figures and Tables

**Table 1:** Demographic and clinical characteristics of the study population.

Variables	DBCM Group (*n* = 37)	No-DBCM Group (*n* = 88)	Non-Diabetic Controls (*n* = 42)	*p*-Value
Age (years)	62 ± 6	60 ± 8	63 ± 5	0.115
Men (*n*)	22 (59.5%)	48 (54.5%)	25 (59.5%)	0.981
Hypertension (*n*)	25 (69.44%)	47 (53.40%)	25 (59.5%)	0.300
Dyslipidemia (*n*)	27 (75%)	61 (72.61%)	29 (69%)	0.787
Smoking (*n*)	10 (27.77%)	20 (23.80%)	11 (26.19%)	0.645
BMI (kg/m^2^)	29.86 ± 9.17	29.88 ± 9.15	28.56 ± 4.55	0.088
SBP (mmHg)	148 ± 17	143 ± 13	138 ± 7	0.102
DBP (mmHg)	88 ± 7	89 ± 8	85 ± 8	0.702
RCAVI (m/s)	8.45 ± 1.14	7.78 ± 1.21	6.86 ± 1.07	0.026 ^a,b,c^
LCAVI (m/s)	8.56 ± 1.21	7.86 ± 1.24	6.90 ± 1.04	0.022 ^a,b,c^
CAVI (m/s)	8.51 ± 1.17	7.82 ± 1.22	6.88 ± 1.05	<0.001 ^a,b,c^
BNP (pg/mL)	91.48 ± 50.69	35.10 ± 18.45	29.79 ± 11.63	<0.001 ^a,b^
Troponin (ng/mL)	3.94 ± 1.02	2.43 ± 1.01	2.07 ± 1.37	<0.001 ^a,b^
Galectin				

BMI, Body Mass Index; BNP, B-natriuretic peptide; CAVI, cardio-ankle vascular index; LCAVI, left cardio-ankle vascular index; RCAVI, right cardio-ankle vascular index; DBCM, Diabetic cardiomyopathy; DPB, Diastolic blood pressure; *n*, number of patients; SBP, systolic blood pressure. Continuous variables are presented as mean ± SD. ^a^ DBCM vs. no DBCM *p* < 0.050; ^b^ DBCM vs. non-diabetic controls *p* < 0.050; ^c^ No DBCM vs. non-diabetic controls *p* < 0.050.

**Table 2:** Results of resting echocardiography and diastolic stress echocardiography at baseline.

Parameters	DBCM Group (*n* = 37)	No-DBCM Group (*n* = 88)	Non-Diabetic Controls (*n* = 42)	*p*-Value
LVEF (%)	63 ± 5	63 ± 5	63 ± 7	0.968
E/A	0.931 ± 0.217	0.931 ± 0.298	0.975 ± 0.298	0.799
E/e′	8.64 ± 2.31	7.59 ± 1.91	7.01 ± 1.1	0.020 ^a,b^
TAPSE (cm)	2.47 ± 0.422	2.31 ± 0.39	2.68 ± 0.532	0.069
TRVmax (m/s)	2.21 ± 0.31	2.05 ± 0.32	2.11 ± 0.44	0.274
RV S’ (m/s)	0.14 ± 0.016	0.14 ± 0.029	0.15 ± 0.015	0.830
LAVI (mL/m^2^)	39.71 ± 8.27	33.46 ± 9.50	31.11 ± 5.60	0.012
SV (mL)	60.30 ± 19.05	71.77 ± 19.84	75.13 ± 16.46	0.039
GLS (%)	−18.87 ± 2.65	−18.73 ± 1.50	−19.22 ± 1.12	0.790
GWI (mmHg%)	2197.79 ± 544.30	2042.11 ± 497.11	2055.89 ± 600.84	0.200
GWE (%)	95.66 ± 2.74	95.47 ± 3.02	95.91 ± 3.05	0.810
GWW (%)	80.58 ± 36.32	91.68 ± 44.20	90.11 ± 44.20	0.573
GCW (mmHg%)	2267.26 ± 333.21	2232.73 ± 361.14	2309.95 ± 580.23	0.714
E/A exercise	1.05 ± 0.17	1.17 ± 0.39	1.21 ± 0.25	0.075
E/e′ exercise	10.82 ± 3.59	8.50 ± 2.35	7.02 ± 1.77	<0.001 ^a,b,c^
TRVmax exercise (m/s)	3.09 ± 0.5	2.35 ± 0.35	2.31 ± 0.44	<0.001 ^a,b^
DTRVmax (m/s)	0.88 ± 0.2	0.3 ± 0.11	0.3 ± 0.12	<0.001 ^a,b^
HFA-PEFF score: 2–4 at rest (*n*)	9	75	-	-
HFA-PEFF score: 0–1 at rest (*n*)	0	13	42	-
Added points to final HFA-PEFF score from DSTE (*n*)	0 points:11 1 points:23	-	-	-

DBCM, diabetic cardiomyopathy; DSTE, diastolic stress echocardiography; DTRVmax: Maximum tricuspid regurgitation velocity during diastole; E/A: Ratio of early (E) to late (A) diastolic mitral inflow velocities; E/e′ exercise: Ratio of early diastolic mitral inflow velocity (E) to early diastolic mitral annular velocity (e′) measured during exercise; GCW, global contractive work; GLS, global longitudinal strain; GWE, global work efficiency; GWI, global work index; GWW, global wasted work; HFA-PEFF, Heart Failure Association Pre-test assessment, Echocardiography and natriuretic peptide, Functional testing, Final etiology; LVEF, left ventricular ejection fraction; LAVI, left atrium volume index; *n*, number of patients RV S’, right ventricular systolic velocity; SD, standard deviation; SV, stroke volume; TAPSE, tricuspid annular plane systolic excursion; TRV, tricuspid regurgitant velocity. Results are presented as Mean ± SD for continuous variables. ^a^ DBCM vs. No DBCM *p* < 0.050; ^b^ DBCM vs. non-diabetic controls *p* < 0.050; ^c^ No DBCM vs. non-diabetic controls *p* < 0.050.

**Table 3:** Results of CAVI, resting echocardiography, and diastolic stress echocardiography (DSTE) at the end of the study.

Parameters	DBCM Group (*n* = 36)	No-DBCM Group (*n* = 86)	*p*-Value
LVEF (%)	64 ± 6	64 ± 5	0.919
E/A	0.95 ± 0.27	0.96 ± 0.32	0.852
E/e′	8.48 ± 2.57	7.78 ± 2.99	0.081
TAPSE (cm)	2.31 ± 0.51	2.40 ± 0.42	0.382
TRVmax (m/s)	2.3 ± 0.3	2.1 ± 0.3	0.368
RV S’ (m/s)	0.13 ± 0.02	0.14 ± 0.03	0.397
LAVI (mL/m^2^)	37.98 ± 8.04	33.11 ± 8.12	0.049
GLS (%)	−18.38 ± 2.11	−18.88 ± 2.66%	0.398
GWI (mmHg%)	2087.82 ± 456.20	2101.07 ± 589.12	0.511
GWE (%)	95.13 ± 3.25	95.89 ± 3.88	0.789
GWW (%)	89.85 ± 47.21	85.24 ± 41.25	0.676
GWC (mmHg%)	2221.25 ± 391.15	2318 ± 398.41	0.487
E/A exercise	1.11 ± 0.13	1.09 ± 0.21	0.354
E/e′ exercise	10.55 ± 4.03	8.10 ± 3.56	<0.001
TRVmax exercise (m/s)	2.95 ± 0.37	2.31 ± 0.20	<0.001
HFA-PEFF score: 0–1 at rest (*n*)	0	12	<0.001
HFA-PEFF score: 2–4 at rest (*n*)	7	65	<0.001
Added points to final HFA-PEFF score from DSTE (*n*)	0 points: 12 1 points: 21 2–3 points: 3	0 points: 84 1 points: 2 2–3 points: 0	-
TRVmax exercise ≥ 2.9 m/s (*n*)	29	7	-

Abbreviations: DBCM, diabetic cardiomyopathy; DSTE, diastolic stress echocardiography; E/A: Ratio of early (E) to late (A) diastolic mitral inflow velocities; E/e′ exercise: Ratio of early diastolic mitral inflow velocity (E) to early diastolic mitral annular velocity (e′) measured during exercise; GCW, global contractive work; GLS, global longitudinal strain; GWE, global work efficiency; GWI, global work index; GWW, global wasted work; HFA-PEFF, Heart Failure Association Pre-test assessment, Echocardiography and natriuretic peptide, Functional testing, Final etiology; LVEF, left ventricular ejection fraction; LAVI, left atrium volume index; RV S’, right ventricular systolic velocity; SD, standard deviation; TAPSE, tricuspid annular plane systolic excursion; TRV, tricuspid regurgitant velocity. Results are presented as Mean ± SD for continuous variables.

**Table 4:** Pharmaceutical changes.

Medications	DBCM Group (*n* = 36)		No-DBCM Group (*n* = 86)	
	Baseline	End	Baseline	End
ACEIs/ARBs	21	28	44	51
CCBs	13	12	24	26
Diuretics	5	14	2	2
Statins	27	28	61	63
SGLT-2 inhibitors	4	33	6	8

Abbreviations: DBCM, diabetic cardiomyopathy; *n*, number of patients; ACEIs, angiotensin-converting enzyme inhibitors; ARBs, angiotensin receptor blockers; CCBs, calcium channel blockers; SGLT-2, sodium-glucose co-transporter 2.

**Table 5:** Univariate and multivariate logistic regression analysis of DBCM presence in our diabetic cohort.

Variable	Univariable		Multivariable	
	OR (95% CI)	*p*	OR (95% CI)	*p*
E/e′	1.40 (0.1.18–1.91)	0.030	1.15 (1.02–1.58)	0.673
CAVI	2.11 (1.51–2.75)	0.007	1.91 (1.69–2.42)	0.042
Troponin	1.25 (1.01–2.58)	0.040	1.05 (1.01–2.21)	0.532
BNP	7.55 (4.28–11.78)	<0.001	5.45 (3.81–9.02)	0.006
DSTE-TRVmax	10.41 (12.07–20.88)	<0.001	8.56 (6.62–11.63)	<0.001
DSTE-E/e′ ratio	1.60 (0.95–2.25)	0.048	1.15 (1.03–1.45)	0.342

Abbreviations: BNP, B-type natriuretic peptide; CAVI, cardio ankle vascular index; CI: confidence intervals; DSTE, diastolic stress echocardiography; E/e′ exercise: Ratio of early diastolic mitral inflow velocity (E) to early diastolic mitral annular velocity (e′) measured during exercise; OR, odds ratio; TRVmax, tricuspid regurgitant velocity.

## Data Availability

The literature cited in this review article was sourced from MEDLINE and EMBASE, Web of Science, Cochrane, and Google Scholar databases. All referenced publications are publicly available through these databases, ensuring accessibility and transparency in data availability.

## Abbreviations

The following abbreviations are used in this manuscript:

BNP	B-type natriuretic peptide
BP	Blood pressure
BMI	Body mass index
CAVI	Cardio-Ankle Vascular Index
DBCM	Diabetic cardiomyopathy
DSTE	Diastolic stress echocardiography
GCW	Global constructive work
GLS	Global longitudinal strain
GWE	Global work efficiency
GWI	Global myocardial work index
GWW	Global wasted work
HF	Heart failure
HFpEF	HF with preserved ejection fraction
HFrEF	HF with reduced ejection fraction
HFA-PEFF	Heart Failure Association Pre-test Probability of HFpEF
HsTn	High-sensitivity troponin
IQR	Interquartile range
LA	Left atrium
LAVI	Left atrium volume index
LV	Left ventricle
LVEF	Left ventricular ejection fraction
LVFP	Left ventricular filling pressures
LVMI	left ventricular mass index
MW	Myocardial work
PWV	Pulse wave velocity
RV	Right ventricle
STA	Speckle tracking analysis
SD	Standard deviation
TAPSE	Tricuspid annular plane systolic excursion
TRVmax	Tricuspid regurgitation velocity maximum
